# Chrysophanol, Physcion, Hesperidin and Curcumin Modulate the Gene Expression of Pro-Inflammatory Mediators Induced by LPS in HepG2: In Silico and Molecular Studies

**DOI:** 10.3390/antiox8090371

**Published:** 2019-09-03

**Authors:** Nabil Mohamed Selim, Abdullah Abdurrahman Elgazar, Nabil Mohie Abdel-Hamid, Mohammed Rizk Abu El-Magd, Aziz Yasri, Hala Mohamed El Hefnawy, Mansour Sobeh

**Affiliations:** 1 Department of Pharmacognosy, Faculty of Pharmacy, Cairo University, Cairo 12613, Egypt; 2 Department of Pharmacognosy, Faculty of Pharmacy, Kafrelsheikh University, Kafrelsheikh 33516, Egypt; 3 Department of Biochemistry, Faculty of Pharmacy, Kafrelsheikh University, Kafrelsheikh 33516, Egypt; 4 Department of Anatomy, Faculty of Veterinary Medicine, Kafrelsheikh University, Kafrelsheikh 33516, Egypt; 5 AgroBioSciences Research Division, Mohammed VI Polytechnic University, Lot 660–Hay MoulayRachid, Ben-Guerir 43150, Morocco; 6 Institute of Pharmacy and Molecular Biotechnology, Heidelberg University, Im Neuenheimer Feld 364, 69120 Heidelberg, Germany

**Keywords:** p38 MAPK, phenolics, hepatoprotective, in silico screening, molecular docking

## Abstract

Hepatitis is an inflammatory condition that can develop hepatocellular carcinoma. Traditional medicine has always been the pillar of medical practice. However, it became less compatible with the current understanding of the diseases and the possible treatment. Therefore, in silico tools could be utilized for building the bridge between the legacy of the past and the current medical approaches allowing access to new therapeutic discoveries. In this work, a Chinese traditional medicine database was screened using structure-based virtual screening to identify molecules that could inhibit p38 alpha mitogen-activated protein kinase (MAPK). Out of the identified compounds, four selected compounds: chrysophanol, physcion, curcumin and hesperidin were isolated from their respective sources and their structures were confirmed by spectroscopic methods. These compounds decreased the gene expression of tumor necrosis factor-alpha (TNF-α), interleukin-6 (IL-6) and interleukin-1beta (IL-1β) in lipopolysaccharide (LPS) induced inflammation in a hepatocellular carcinoma cell line (HepG2) in a dose-dependent manner. The molecular docking study revealed the specificity of these compounds towards p38 MAPK rather than other MAPKs. In conclusion, the molecular and in silico studies suggest that the isolated compounds could be a potential treatment for hepatitis by resolving inflammation controlled by MAPKs, thus limiting the development of further complications and lower side effects.

## 1. Introduction

Liver is the central organ responsible for detoxification and metabolism in the body. The impairment of hepatic function can cause serious complications which may lead finally to death. Hepatic disorders are a major generic health obstacle in Egypt, where it bears the highest prevalence rate in the world [1]. There are several factors that may cause liver failure, among them drugs, alcohol, toxins and viral infections. However, the predominant pathway of pathogenesis is mediated by inflammation [2].

The mitogen activated protein kinase (MAPK) pathway has an essential role in modifying external stimuli, intercellular responses and several post transcriptional factors that control inflammation, cell differentiation and apoptosis. MAPKs are divided into, at least, 5 major subfamilies namely p38 MAPKs, Jun N-terminal kinases (JNKs), extracellular signal-regulated kinase 1 and 2 (ERK1/2), and ERK5. It has been established that this family of proteins has predominant role in the inflammation, fibrogenesis and carcinogenesis of hepatocytes, thus becoming an essential target for effective prevention and treatment of various liver diseases [3,4,5].

The continuous searching and development of hepatoprotective and anti-fibrotic agents has become of great importance. Several natural compounds derived from traditional medicine were reported to possess hepatoprotective effect [6,7,8]. However, there is lack in studies addressing the exact molecular mechanism behind this effect, which is a common challenge in the natural products research [9]. Therefore, molecular docking studies can explain the interactions between phytochemicals and the molecular targets, which could be a successful approach to develop new drugs.

Utilizing high throughput virtual screening, 60,000 secondary metabolites derived from natural sources were investigated for their possible inhibition of the MAPK pathway in our former work [10]. In this study, out of the top hundred active hits, we isolated 4 secondary metabolites from their respective sources: namely curcumin from *Curcuma longa* L. *rhizomes*, hesperidin from *Citrus sinensis* L. fruits, chrysophanol and physcion from *Rhubarb officinale*, L. rhizomes. We explored the role of the representative compounds to modulate the expression of pro-inflammatory mediators induced by lipopolysaccharide (LPS) in HepG2 cell lines. Also, we addressed how these compounds interact with MAPKS utilizing molecular docking tools.

## 2. Materials and Methods

### 2.1. Equipment

A double beam UV/Vis spectrophotometer (t80+ (PG instruments Ltd., Leicestershire, UK)), and infra-red spectrophotometer (Thermo Scientific Nicolette iSTM10 FT-IR spectrometer) were used. Thermo scientific ISQLT single quadrupole (USA) mass spectrometer was used to determine the MS data of the isolated compounds. Tetramethylsilane TMS was used as an internal standard to record nuclear magnetic resonance spectra (^1^H NMR, ^1^H NMR, ^13^C NMR) utilizing BRUKER Ascend^TM^ 400 spectrometers operating at 400 MHz. Quantitative PCR was utilized using step one plus real time thermal cycler (Applied Biosystems, Life Technology, Foster, CA, USA).

### 2.2. Plant Material

Turmeric (*Curcuma longa* L, family Zingiberacea) and Rhubarb powder (*Rhubarb officinale* L, family Polygonacea) were obtained from Alpha-Chemika. (Mumbai, India). Orange fruits were purchased from local market in Kafrelsheikh Governorate and authenticated by Prof. Abd El Halim A. Mohamed, taxonomist at the Agricultural Museum, Dokki, Cairo. A voucher specimen was deposited at the herbarium of Pharmacognosy Department, Faculty of Pharmacy, Cairo University under number (#2015.06.16 b.). The fruits were peeled off and the peels were dried under shade, pulverized to give fine powder (300 g).

### 2.3. Extraction and Isolation

#### 2.3.1. Isolation of Curcumin from Turmeric

Defatted 450 g of turmeric powder, with hexane, was exhaustively extracted using acetone, which was concentrated and dried under vacuum to give 30 g. The latter was extracted again using hot ethanol to yield 15 g yellow powder of total curcuminoids.

Five grams of this fraction were applied on a flash column chromatography with polar silica gel (80 g) using gradient elution beginning with 100% dichloromethane, then a continuous increase in polarity up to 6% methanol with a flow rate 34 mL/min. Fractions were monitored with the aid of photodiode array (PDA) detector and the similar ones were gathered together based on thin layer chromatography (TLC) investigation to give compound **1**.

#### 2.3.2. Isolation of Anthraquinones from Rhubarb

Rhubarb powder (300 g) was subjected to acid hydrolysis using 10% HCl for 2 h, to increase the amount of free aglycones, then filtered and dried before extraction with methylene chloride till exhaustion. The extract was evaporated under vacuum and subjected to liquid-liquid fractionation using 10% NaHCO_3_ and concentrated to give 2 g of yellowish-brown powder. Five hundred milligrams of this fraction were applied on 25 g normal phase flash column chromatography. Elution was carried out using step gradient system of (a) hexane: (b) toluene: (c) methylene chloride, starting with isocratic elution using ratio A: B (89:11) for 2 column volumes (CV), followed by increase in B to 42% over 15 (CV). Finally, methylene chloride was added to the gradient with continuous decrease in A to 0% and increase in C to 60% over 15 (CV), using flow rate 15 mL/min. Fractions were combined based on (PDA) detector and TLC (pre-coated silica gel 60 GF254 (20 × 20 cm, 0.2 mm thick, Merck, Germany) screening to give compound **2** and **3**.

#### 2.3.3. Isolation of Hesperidin from Citrus Fruits

One liter of petroleum ether was added to 250 g of dried powder of orange peel, heated for 4 h under reflux and the extract was discarded. The peels were re-extracted using methanol for 2 h under reflux, filtered and evaporated under vacuum till syrup consistency. 50 mL of 6% acetic acid was added to the residue to precipitate solid crude product and then washed by 6% acetic acid and dried at 60 °C for further purification. The crude product was dissolved in dimethyl sulfoxide (DMSO) under stirring with addition of water and heated at 60–80 °C. Hesperidin precipitates after cooling, sucked off and washed with little warm water and subsequentially with iso-propanol, then dried in the desiccator, yielding 2.5 g of white powder (compound **4**).

The isolated compounds (**1**–**4**) were characterized by comparing their spectroscopic data (IR, ^1^H NMR, ^13^C NMR, and MS) with previously published in the literature.

### 2.4. Cell Culture and Cytotoxicity Study

HepG-2 cells, purchased from VACSERA, Cairo, Egypt, were seeded in Dulbecco’s Modified Eagle Medium (DMEM) [supplemented with 10% (*v*/*v*) fetal calf serum (FCS, Hyclone, Logan, UT, USA) and 1% (*v*/*v*) penicillin/streptomycin solution (Hyclone, Logan, UT, USA)] at 37 °C in 5% CO_2_ (CO_2_ incubator (SHEL LAB, Sheldon Manufacturing NC., Cornelius, OR, USA). The medium was changed every three days and passaged at 80% confluence after trypsinization (0.05%, *w*/*v*). Cancer cells were cultured at a density of 1 × 10^4^ cells/well (100 μL/ well). The tested compounds were added in triplicate separately to the wells to obtain final concentrations of 100 μg/mL up to 3.125 μg/mL and incubated for 24 h. A solution of MTT [3-(4,5-dimethylthiazol-2-yl)-2,5-diphenyltetrazolium bromide] (5 mg/mL) in sterile PBS was applied to each well and the plate was incubated for 4 h at 37 °C. The solution was discarded, and the insoluble formazan crystals were dissolved using 100 μL of DMSO and left for 20 min. The plate was read using plate reader at λ_max_ 570 nm. From the constructed dose response curve. The IC_50_ was expressed as the concentration of the compounds that inhibit 50% of cells

### 2.5. Induction the Production of Inflammatory Markers in HepG2

HepG2 cells were pre-cultured in serum-free DMEM for at least 4  h to minimize mitogenic effects [11]. The plate was divided according the following design: control cells treated by vehicle (DMSO), cells treated by inflammatory inducer [1 µg/mL LPS from *E. coli*, Sigma Aldrich (St. Louis, MO, USA)], and cells treated by different concentrations (0.1, 1, 5 and 10 µg/mL) of tested compounds in triplicate, respectively before stimulation with LPS by 1 h. Finally, the plate was incubated for 24  h.

### 2.6. Quantitative Real Time PCR Analysis

The isolation of total RNA from the cells was performed using RNeasy Mini kit (# 74104, Qiagen, Germany) containing DNase I following manufacturer’s guidelines as previously described [12]. cDNA was constructed from 4 mg of the isolated mRNA using reverse transcriptase kits and used for determination of the relative expression of the genes under investigation in the cell line. The concentration of isolated mRNA and cDNA were determined utilizing Nanodrop spectrophotometer Q5000/USA. Beta-actin, the housekeeping gene, was used as an internal standard. The prepared cDNA was magnified using 2X Maxima SYBR Green/ROX qPCR Master Mix as described in the manufacturer guidelines (Thermo scientific, USA, # K0221) and the primers for the selected genes. The primers applied in the amplification are shown in Table 1. They were developed using primer 3 online interface [13]. PCR conditions were done as previously described [14]. The melt curve was produced by increasing temperature from 60 to 95 °C. The relative variation in gene expression was calculated based on fold change derived from quantities critical threshold (Ct) using 2^-∆∆Ct^ method [15].

### 2.7. In Silico Study for the Interaction of the Identified Compounds with MAPKs.

Mitogen activated protein kinases (p38, ERK2, JNK1) and MK-3 were chosen for in silico study, their 3D structures were obtained from protein data bank (www.pdb.org), using the following PDB-code (p38α: 2QD9; ERK2: 5NHF; JNK1: 1UKI; MK-3: 3SHE). The 3D structure of compounds under investigation was regained from Zinc Database (http://zinc.docking.org) as mol2 file format.

### 2.8. Preparation of Active Site for Molecular Docking

The interaction between the co-crystallized ligand and the amino acids residue of the active site was used to recognize the binding site using active site preparation module in LeadIT software (https://www.biosolveit.de/LeadIT/). The binding site was defined as 6.5 ˚A around the co-crystallized ligand. Since water molecules have no role in this type of interaction, they were removed from calculation.

#### Molecular Docking of the Isolated Compounds in MAPKs Active Site

Molecular docking module FlexX in LeadIT software was chosen for our study [16]. The establishment of this module was confirmed by redocking the reference ligand in the active site of the target and assessing the ability of the software to reproduce the pose proven experimentally. Default docking parameters were used. Compounds were ranked according to their binding energy. Compounds ranked in the top ten were chosen for rescoring and forecast of the estimated *K_i_* by HYDE scoring function. The best poses were selected for visual inspection in molecular operating environment to determine the putative binding mode.

### 2.9. Statistical Analysis

One-way analysis of variance (ANOVA) was used for estimation of statistically significant values using SPSS, 18.0 software, 2011. Values were considered statistically significant when *p* < 0.05. The results were expressed as means ± S.E. and the comparisons were acquired individually by Duncan’s multiple range test (DMRT).

## 3. Results and Discussion

### 3.1. Identifictation of the Isolated Compounds (1–4)

**Figure 1:**
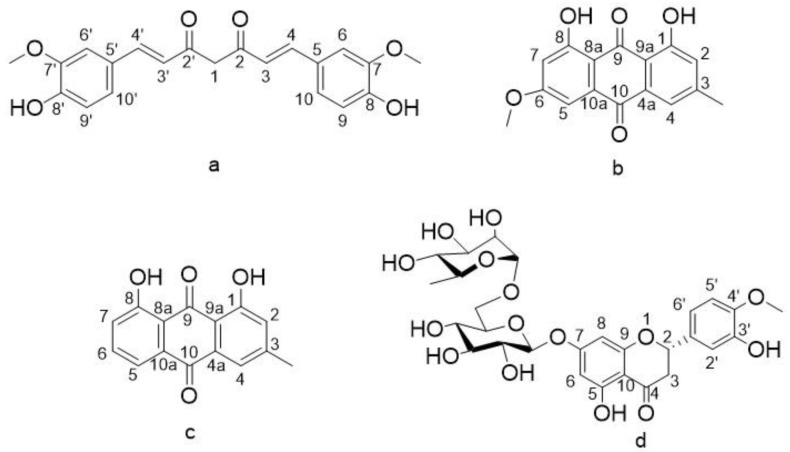
Chemical structures of the isolated compounds: (**a**) Curcumin, (**b**) Physcion, (**c**) Chrysophanol and (**d**) Hesperidin.

**Compound 1**: Orange red needles, 250 mg, m.p. 183 °C, exhibited a molecular ion at *m/z* 368 *calc.* for C_21_H_20_O_6_. IR: 3610, 1627, 1512, 1280 cm^−1^, ^1^H NMR δ (ppm) DMSO-*d6*, 400 MHz: 9.66 (2H, s, 8,8 OH), 7.5 (2H, d, J = 16Hz, H-5, 5’), 7.32 (2H, s, H-6,6’), 7.16 (2H, d, J =8 Hz, H-10,10’), 6.82 (2H, d, J = 8Hz, H-9, 9’), 6.73 (2H, d, J = 16Hz, H-3, 3’), 6.065 (1H, s, H-1), 3.9 (6H, s, H-7 and 7’ OCH_3_). ^13^C NMR, 100 MHz δ (ppm): 183.27 (C-2, 2’), 147.88, (C- 8, 8’), 146.82 (C- 7,7’), 140.56 (C- 4,4’), 127.7 (C- 5, 5’), 122.88 (C- 10, 10’), 121.79 (C- 3, 3’) 114.86 (C- 9, 9’), 109.69 (C- 6, 6’), 101.15 (C- 1), 55.99 (C- OCH_3_). After comparison of these data with the reported studies, this compound was confirmed to be curcumin (Figure 1a) [17].

**Compound 2:** Orange yellow needle, 150 mg, m.p. 206–207 °C, respond to Borntrager’s test giving rose red color and have a molecular ion at *m/z* 284.27 *calc*. for C_16_H_12_O_5_; IR: 3423, 1665, 1625, 1570 cm^−1^, ^1^H NMR δ (ppm) CDCl_3_, 400 MHz: δ12.34 (1H, s, 1-OH), 12.14 (1H, s, 8-OH), 7.65 (1H, s, H-4), 7.39 (1H, brs, H-2), 7.11 (1H, s, H-5), 6.72 (1H, brs, H-7), 3.97 (3H, s, OCH_3_), 2.49 (3H, s, CH_3_). ^13^C NMR, 100 MHz δ (ppm): 190.78 (C-9), 181.98, (C-10), 166.54 (C-6), 165.18 (C-8), 162.50 (C-1), 148.43 (C-3), 135.25 (C-10a) 130.86 (C-4a), 124.49 (C-2), 121.27 (C-4), 121.27 (C-5), 1113.67 (C-9a), 108.19 (C-8a), 106.76 (C-7), 56.07 (OCH_3_), 22.15 (CH_3_). Compound 2 was recognized as physcion according to the compared spectroscopic data (Figure 1b) [18,19].

**Compound 3:** Orange yellow plates, 200 mg, m.p. 193–194 °C, respond to Borntrager’s test giving rose red color and a molecular ion at *m/z* 254.06 *calc*. for C_15_H_10_O_4_; IR: 3435, 1676, 1627, 1568 cm^−1^, ^1^H NMR δ (ppm) CDCl_3_, 400 MHz: 12.7 (1H, s, 8-OH), 11.95 (1H, s, 1-OH), 7.77 (1H, brd, J = 7.5 Hz, H-5), 7.63 (1H.t, J = 7.6 Hz, H-6), 7.59 (1H, brs, H-4), 7.25 (1H, brd, J = 8.5Hz, H-7) 7.05 (1H, brs, H-2), 2.44 (3H, s, CH_3_). ^13^C NMR, 100 MHz δ (ppm): 192.4 (C-9), 181.85 (C-10), 162.5 (C-1), 162.2 (C-8), 149.3 (C-3), 136.9 (C-6), 133.5 (C-10a) 133.2 (C-4a), 124.5 (C-7), 124.32 (C-2), 121.3 (C-4), 119.89 (C-5), 115.8 (C-8a), 113.66 (C-9a), 22.24 (CH_3_). The structure of this compound was determined as chrysophanol after comparing it with the spectral data from the previous studies (Figure 1c) [20,21].

**Compound 4:** white powder, 2.5 g, m.p. 262–263 °C, showing a molecular ion at *m/z* 610 *calc*. for C_28_H_34_O_15_, λmax (MeOH) nm: 328, 282.8, 225.8 and; IR: 3417, 3089, 2935, 1693 and 1647 cm^−1^, ^1^H NMR δ (ppm) DMSO-d6, 400 MHz: (aglycone) 12.01 (1H, br s, 5-OH), 6.96 (1H, brs, H-2′), 6.95 (1H, J = 5.5,2.8 Hz, H-6′), 6.91(1H, dd, J = 8.4, H-5′), 6.15 (1H, d, J = 2.1 Hz, H-8), 6.13 (1H, d, J = 2.5 Hz, H-6), 5.49 (1H, dd, J = 7.8, 3.4 Hz, H-2), 4.98 (1H, d, J = 8 Hz, H-1 glucose), 4.54 (1H, brs, H-1 rhamnose), 3.78 (3H, s, 4-OCH_3_), 3.34 (1H, m, H-3,equaterial), 2.79 (1H, dd, J = 17.0, 5.0 Hz, H-3 axial), 1.09 (3 H, d, J = 6.3 Hz, CH_3_-rhamnose). ^13^C NMR (DMSO-d6, 100 MHz) δ 197.46 (C-4), 165.6 (C-7), 163.49 (C-5), 162.95 (C-9), 148.42 (C-5′), 146.92 (C-4’), 131.17 (C-1′), 118.4 (C-2′), 114.61 (C-6′),112.5 (C-10), 112.2 (C-3′), 103.5 (C-1 glucose), 100.8 (C-1 rhamnose), 99.92 (C-1 rhamnose), 96.84 (C-6), 95.7 (C-8), 78.83(C-2), 66.0 (C-6 glucose), 78.8 – 66.5 (C-2, C-3, C-4 and C-5 glucose and rhamnose), 56.16 (OCH_3_-4’), 17.9 (C-6 rhamnose). From these spectral data and comparison previous reports [22,23], compound 4 was identified as hesperidin (Figure 1d).

### 3.2. Cytotoxic Activity by MTT Method

**Figure 2:**
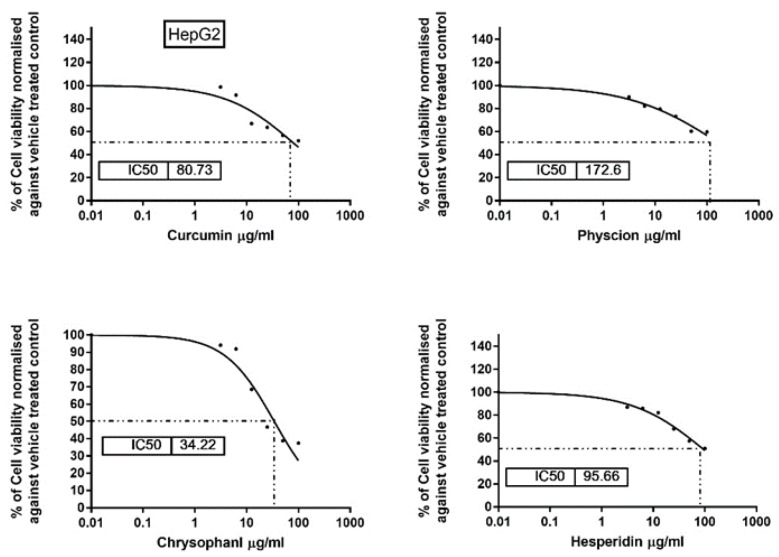
Cytotoxic effect of the isolated compounds on HepG2. Cells were treated with various concentrations of compounds (100, 50, 25, 12.5, and 3.25 µg/mL) for 24 h and the cell viability was determined by MTT assay. The results are expressed as percentage of cell growth relative to negative control (vehicle treated) cells.

MTT assay was performed for determination of subtoxic doses of the isolated compounds. All identified compounds showed moderate cytotoxic activity at high concentration. However, they did not affect the viability of the cells in a concentration up to 20 µg/mL (Figure 2 and Table 2). Therefore, treatment with a concentration up to 10 µg/mL of each compound was safe to be used for all further experiments.

### 3.3. Effect of the Tested Compounds (1–4) on LPS-Induced Gene Expression of Different Inflammatory Mediators

**Figure 3:**
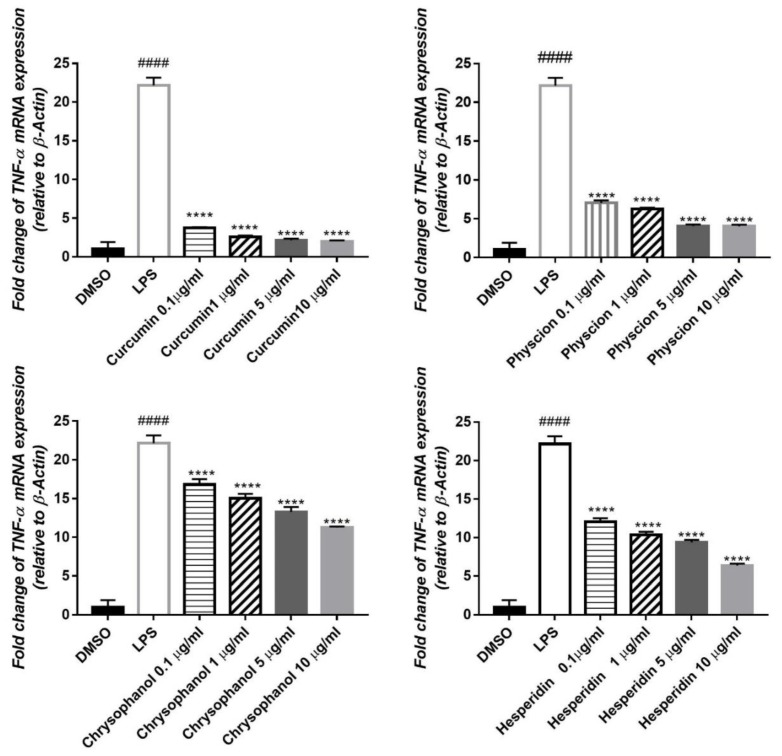
Effect of the isolated compounds (1–4) on the elevated level of *TNF-α* in HepG2 cells induced by 1 µg/mL LPS relative to β-Actin. qPCR was used for estimation of mRNA expression. Results are expressed as ± S.E (*n* = 3), ^####^*p* < 0.0001 compared with control. ****p < 0.0001 compared to LPS.

LPS increased TNFα mRNA expression in the cells to approximately 22 folds. Out of the tested compounds, curcumin restored this value to the normal level. However, physcion, chrysophanol and hesperidin decreased the gene expression up to 18, 11 and 16-fold, respectively (Figure 3).

**Figure 4:**
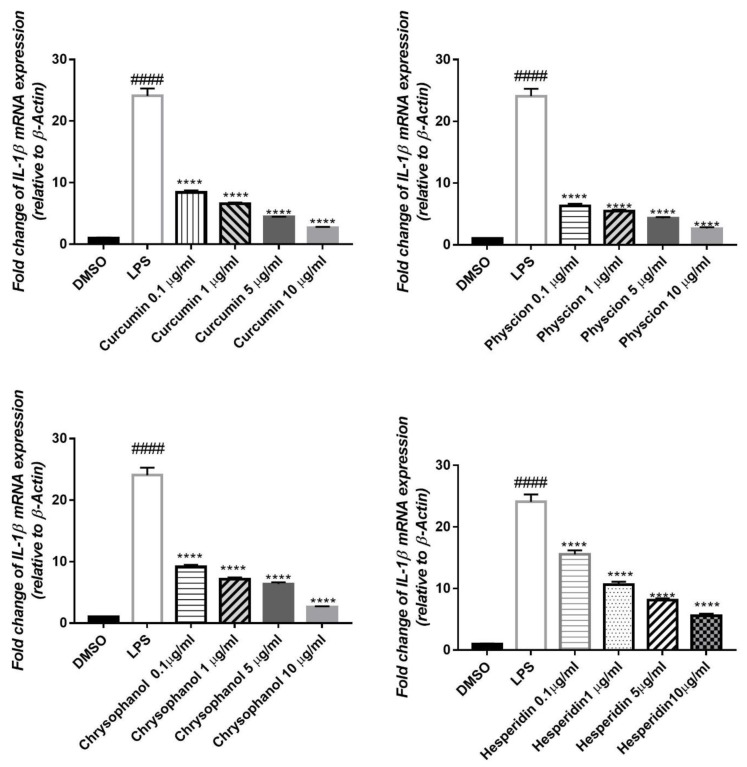
Effects of compounds (1–4) on the elevated level of *IL-1β* expression induced by 1 µg/mL LPS in HepG2, relative to β-Actin. qPCR was used for detection of mRNA expression. Data were given as ± S. E (*n* = 3), ^####^*p* < 0.0001 compared with control. ****p < 0.0001 compared to LPS.

**Figure 5:**
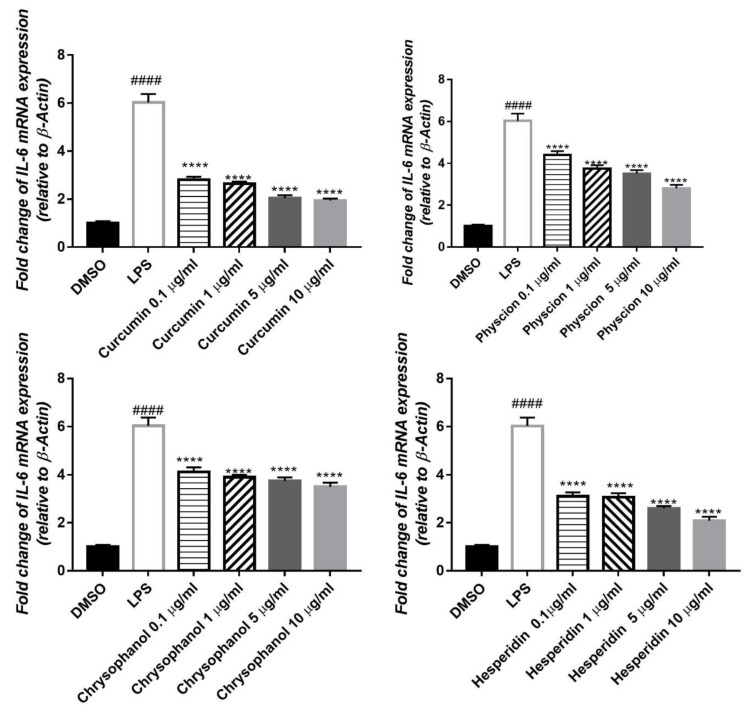
Effects of isolated compounds (1–4) on the elevated level of *IL-6* expression induced by 1 µg/mL LPS in HepG2, relative to β-Actin. qPCR was used for detection of mRNA expression. Results are shown as ± S. E (*n* = 3), ^####^*p* < 0.0001 compared with control. ****p < 0.0001 compared to LPS.

IL-1β mRNA expression was elevated to about 24 folds, by the inflammatory effect of LPS on HepG2 cell lines, compared to the untreated control (DMSO). Pre-treatment with compounds (1–4) significantly reduced the gene expression level in a concentration dependent manner by 21, 21, 21, and 15-fold, respectively (Figure 4). IL-6 mRNA expression in HepG2 was also elevated by the effect of LPS to about 6 folds. Treatment with the isolated compounds (1–4) significantly attenuated this effect in a dose dependent way, where the expression level was decreased by 4, 3.5, 3 and 4, fold, respectively (Figure 5)

### 3.4. In Silico Assessment of the Inhibitory Effect of Isolated Compounds (1–4) on MAPKs

Based on our previous virtual screening results for identifying p38 MAPK inhibitor, 4 main classes of phytochemicals were emerged as potential hits. Redocking of the co-crystallized ligand and analyzing the root mean square deviation value (RMSD) between the experimental and predicted pose was found to be in the acceptable range [10]. In this study, a molecular docking of compounds (1–4) was performed in the active site of the previously mentioned targets to assess their interaction with the key amino acids necessary to achieve an inhibitory effect on MAPKs.

**Figure 6:**
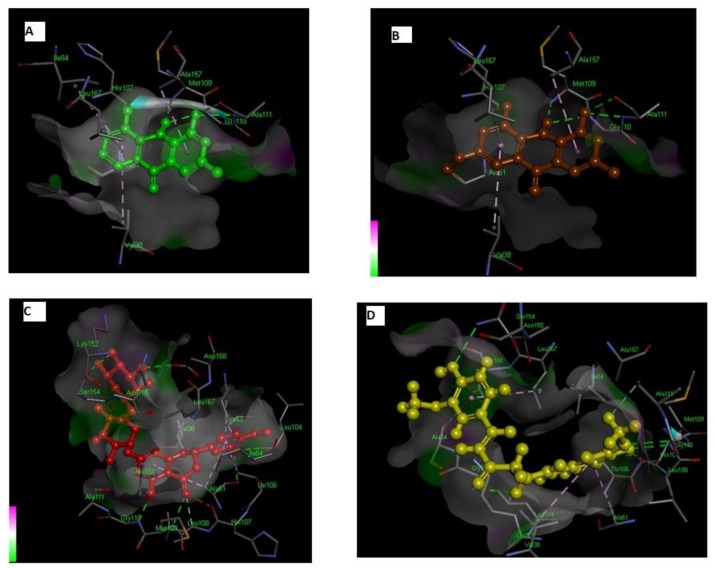
Molecular interactions of chrysophanol (**A**), physcion (**B**), hesperidin (**C**) and curcumin (**D**) with p38 α MAPK (PDB ID: 2QD9).

**Figure 7:**
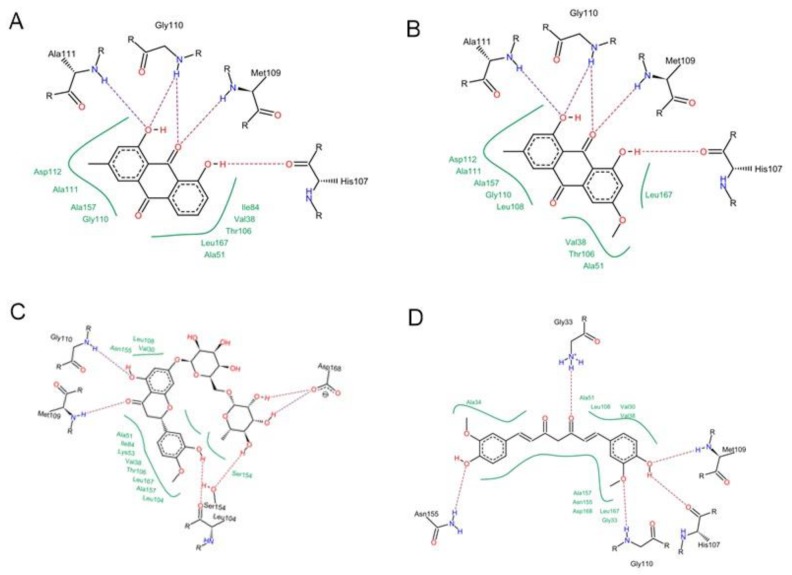
2D Protein-ligand binding diagram of chrysophanol (**A**), physcion (**B**), hesperidin (**C**) and curcumin (**D**) with p38α MAPK (PDB ID: 2QD9).

**Figure 8:**
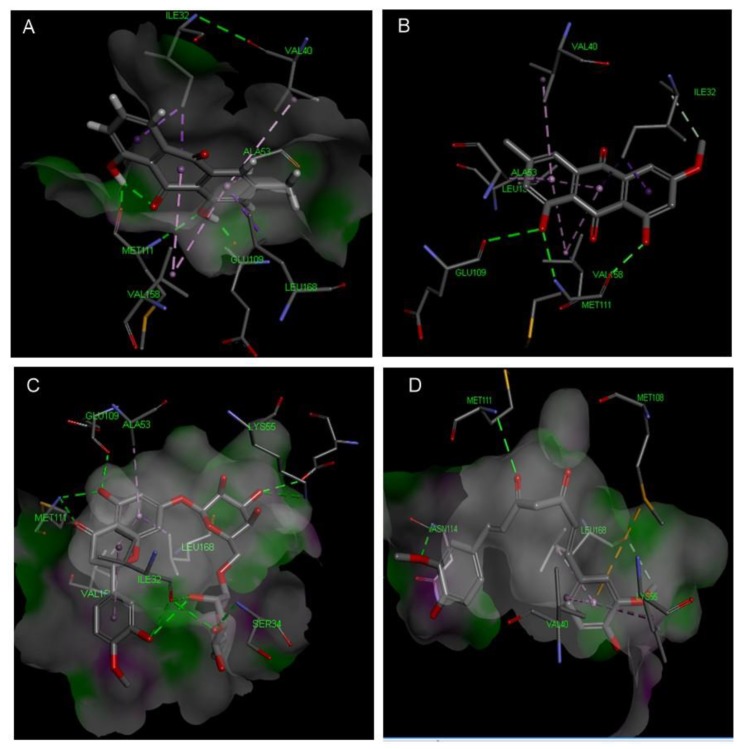
Molecular interactions of chrysophanol (**A**), physcion (**B**), hesperidin (**C**) and curcumin (**D**) with JNK MAPK (PDB ID: 1UKI).

**Figure 9:**
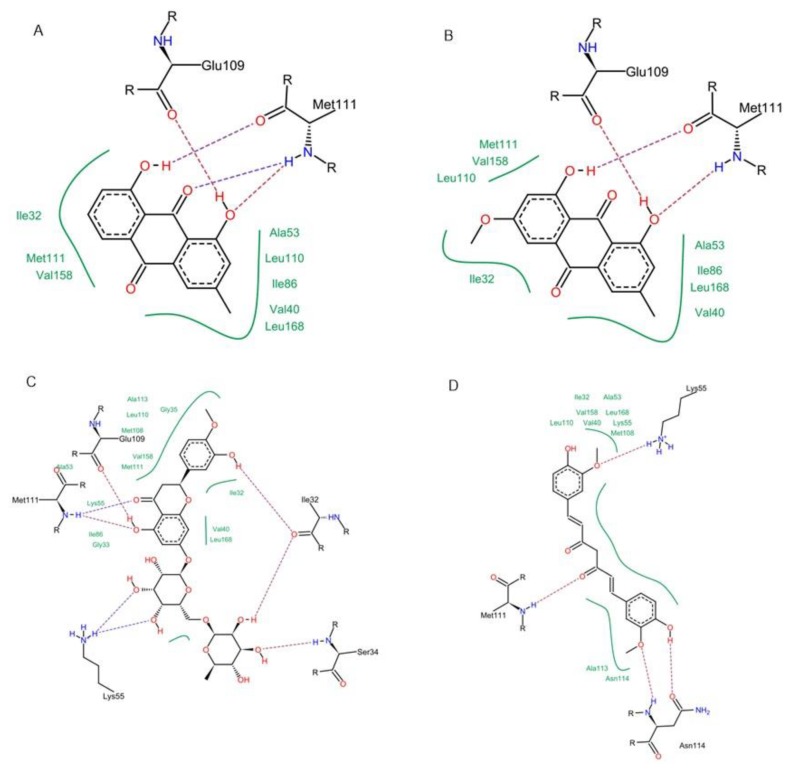
2D Protein ligand binding diagram of chrysophanol (**A**), physcion (**B**), hesperidin (**C**) and curcumin (**D**) with JNK MAPK (PDB ID: 1UKI).

**Figure 10:**
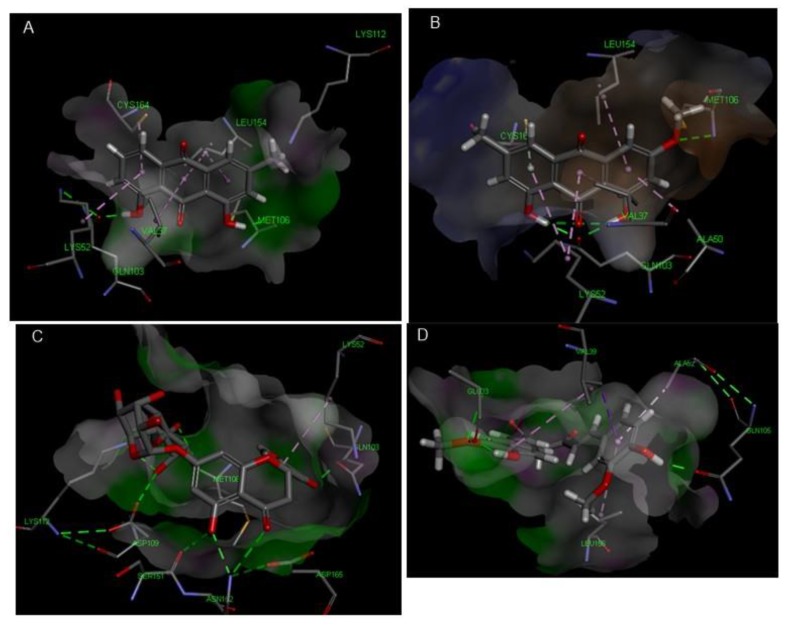
Molecular interactions of chrysophanol (**A**), physcion (**B**), hesperidin (**C**) and curcumin (**D**) with ERK1/2 MAPK (PDB ID: 5NHF).

**Figure 11:**
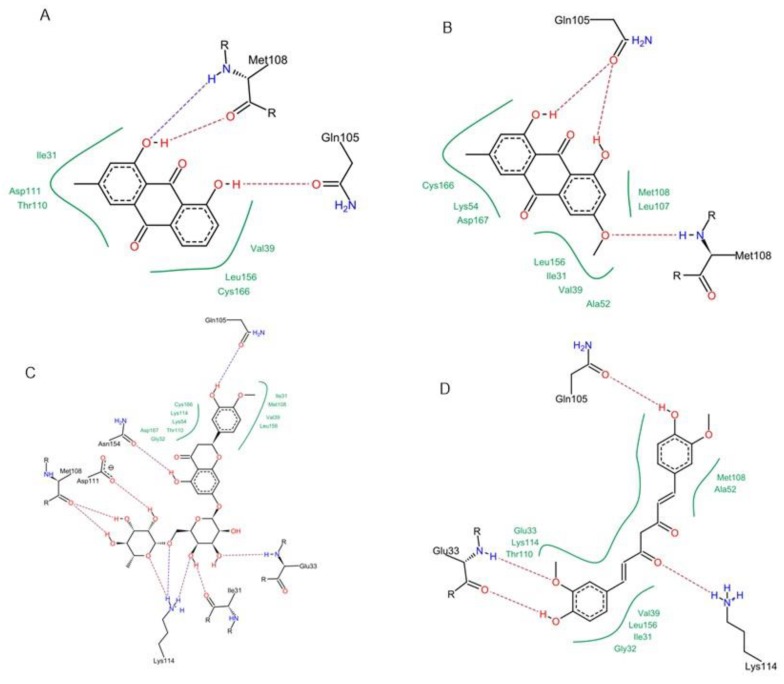
2D Protein-ligand binding interaction diagram of chrysophanol (**A**), physcion (**B**), hesperidin (**C**) and curcumin (**D**) with ERK1/2 MAPK (PDB ID: 5NHF).

**Figure 12:**
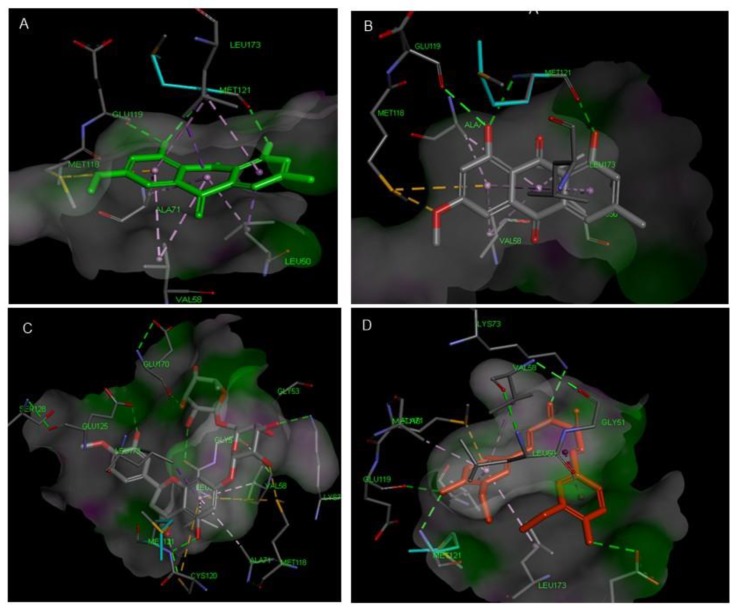
Interactions and binding energy of chrysophanol (**A**), physcion (**B**), hesperidin (**C**) and curcumin (**D**) with MK-3 MAPK (PDB ID: 3SHE).

**Figure 13:**
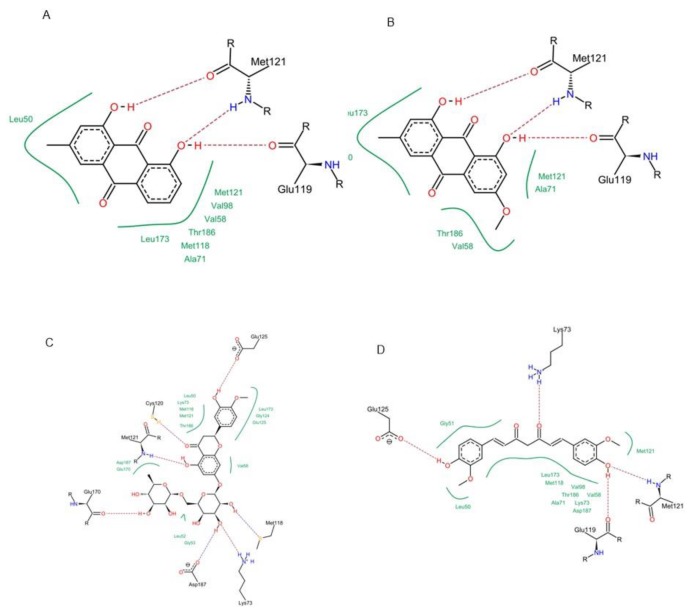
2D Protein-ligand binding interaction diagram of chrysophanol (**A**), physcion (**B**), hesperidin (**C**) and Curcumin (**D**) with MK-3 MAPK (PDB ID: 3SHE).

Concerning p38 α MAPK, all compounds succeeded to fit in the active site properly without any noticed clashes (Figure 6 and Figure 7), and their binding energy was comparable to that calculated for the co-crystallized ligand. HYDE assessment showed that hesperidin has the highest binding affinity. On the contrary, the binding energy of the isolated compounds were higher than the reference molecule for both of JNK1 and ERK1/2, their interactions with the active site are demonstrated in Figure 8, Figure 9, Figure 10 and Figure 11, respectively. The same case was noticed in the analysis of their binding with MK-3, except for curcumin, which possessed a better predicted *K_i_* than of the reference bounded ligand as revealed by HYDE assessment. The docked poses of the compound in active site of MK-3 are shown in Figure 12 and Figure 13. The molecular docking results are summarized in Table 3 including binding energy, predicted *K_i_*, and the interaction of the compounds with amino acid residues in the binding sites of 4 MAPKs (P38 α, ERK2, JNK & MK3). 

The continuous need for novel therapeutic agents requires eminent efforts to open new opportunities from available resources of traditional medicine. This can be achieved only by understanding how their chemical constituents exert their pharmacological actions, which might be a hard task considering the presence of hundreds of compounds in each formula. Fortunately, the emerging of in silico tools has not only accelerated the discovery of novel molecules from natural products but also revealed new activities for known compounds [24,25,26].

Controlling inflammation was found to be a basic strategy for management of several diseases, especially liver diseases. In this context, several attempts were reported to target the MAPK family of enzymes, which have prominent role in liver injury and tumorigenesis [27,28]. Since LPS is a known activator of such pathway, we used it as a tool to investigate the ability of compounds from traditional medicine to attenuate gene expression of some pro-inflammatory markers, such as TNF-α, IL-1β and IL-6, in HepG2 cell line [29,30,31,32].

This model was used to assess the anti-inflammatory effect of several nature derived compounds or fractions based on the fact that hepatic cells are able to express plethora of enzymes and inflammatory markers [33,34], taking in consideration, that all tested compounds should be used in subtoxic doses to maintain the viability of the cells. In this study, all investigated compounds (curcumin, chrysophanol, physcion and hesperidin) were able to reduce the expression of three main pro-inflammatory mediators in LPS-induced inflammation in HepG2 cell lines, in a dose dependent manner of 10 µg/mL as the highest dose.

The off-target related side effects associated with kinase inhibitors is a major concern since the architecture of kinases is very similar, however, the extensive x-ray studies revealed distinguishing features required for achieving selective inhibition. For instance, p38 MAPK inhibition requires the interaction with Met-109, Gly-110 and specificity could be achieved by entering the hydrophobic region including Thr-106, Lys-53, Leu-75, Ala-157, Leu-86, Leu-104, leu-167 and Val-105 [35,36]. The binding with JNK1 requires the interaction with 2 amino acids (MET-111 and GLU-109) but specificity is enhanced by accessing the hydrophobic pocket containing amino acids such as Ile-32, Val-40, Ala-53, Ile-86, Met-108, Leu-110, Val-158, and Leu-168 [37].

The binding in the ATP active site of ERK1/2 requires the interaction with the gate keeper residues (Gln-105), hinge region (Met-108), while selectivity could be achieved by interaction with the so-called glycine rich loop (Tyr-36, Ile-56 and Gly-34) [38]. Finally, for achieving inhibitory effect on MK-3, interactions with the following amino acids is needed: ASP-187, LYS-73, Met-121, Glu-122, Leu-50 and Val-58, however, further interaction with Glu-84, His-88, Glu-125 and Glu-170 is favorable for selectivity [39,40].

The observed anti-inflammatory effects of the isolated compounds were depicted in previous studies [41,42,43,44]. However, there is a scarce in reports addressing their molecular mechanism, especially in case of hepatitis, which we tried to compensate by employing integrated in silico and molecular biology tools. Curcumin, the major diarylheptanoid isolated from Curcuma rhizomes, has wide array of pharmacological activities due to its ability to interact with several therapeutic targets [45]. Our virtual screening predicted its ability to inhibit p38 alpha MAPK phosphorylation by interacting with amino acids residues such as Met-109, Gly-110 and His-107, which are the main requirements for inhibiting the activity of this enzyme.

Interestingly, it was reported that curcumin could downregulate the p38 alpha MAPK in tobacco smoke-induced hepatitis in rats [46]. Curcumin was also able to fit in ATP binding site of ERK1/2 by partial interaction with some of important residue such as Met 108. However, it couldn’t access the so-called glycine-rich loop region, which is important for selectivity. For JNK1, curcumin interacted with MET-111 but not Glu-109, the required residue for binding with most of kinases. It was able to effectively interact with important amino acids such as Glu-119, Lys-73, Met-121 and Thr-186 in the active site of MK-3 which can explain the high binding affinity predicted by HYDE assessment. Molecular docking study suggested that curcumin might exert its action through interacting with p38 and MK-3 rather than other MAPKs, which is in agreement with previous studies stating that “one hydrogen bond acceptor, one hydrogen bond donor and two aromatic rings” are common features for inhibitors of p38 MAP kinase [47].

The two structurally related anthraquinones (chrysophanol and physcion) showed similar behavior in vitro and in silico. On contrary to curcumin, both compounds showed their ability to bind effectively to all of the MAPKs enzymes but with lower extent in case of ERK1/2, which can be explained in the light of the previously mentioned criteria, and is in agreement with former reports indicating the ability of physcion glycoside to exhibit anti-inflammatory effect against rheumatoid arthritis derived fibroblast-like synoviocyte cell line by inhibiting phosphorylation of p38, JNK and ERK MAP kinase [48,49,50]. Yet, there were no studies addressing the ability of anthraquinones to inhibit MK-3. This may suggest that anthraquinone could be a promising scaffold for developing multi-MAP kinase inhibitors. Hesperidin is a flavanone glycoside with prominent pharmacological activities: It’s well known that this class of compounds can bind effectively to the ATP binding site of several kinases especially MAPKs, which explains their reported anti-inflammatory effect by several studies [51], which could be linked to its observed effect on the gene expression of cytokines in our study. Regarding the molecular docking study, hesperidin can interact with ASP-168, Gly-110 and Met-109 by hydrogen bonding. Moreover, the molecule interacted with Ala-157, Thr-106 residues, which are known to be responsible for selective inhibition of p38 alpha rather than other MAPKs [52], thus explaining that hesperidin achieved reasonable binding energy with all of MAPKs especially in case of p38, where it has the highest binding affinity but showed less performance in case of ERK1/2.

## 4. Conclusions

In this study, 4 compounds; curcumin, chrysophanol, physcion and hesperidin were identified as p38 alpha inhibitor by structure based virtual screening; they were isolated in pure form from their respective natural sources and tested for their ability to resolve LPS induced Inflammation in HepG2 cell lines. All compounds reduced gene expression of proinflammatory cytokines such as TNF alpha, interleukin beta one and interleukin 6 in a dose dependent manner. Curcumin and hesperidin exhibited more affinity towards p38 alpha MAPK rather than other kinases, while chrysophanol and physcion could serve as a scaffold for developing more potent MAPKs inhibitors for prevention and treatment of hepatic inflammatory diseases. Structure-activity relationship and *in-vivo* animal models of diseases and safety studies are required before extrapolating these results towards future preclinical investigations.

## Figures and Tables

**Table 1:** Forward and reverse primers sequence for investigated pro-inflammatory genes.

Gene	Forward Primer (‘5 ------ ‘3)	Reverse Primer (‘5 ------ ‘3)
TNF-α	CCCAGGGACCTCTCTCTAATC	ATGGGCTACAGGCTTGTCACT
IL-1β	ACAGATGAAGTGCTCCTTCCA	GTCGGAGATTCGTAGCTGGAT
IL-6	GGTACATCCTCGACGGCATCT	GTGCCTCTTTGCTGCTTTCAC
β-actin	CGACATCAGGAAGGACCTGTATGCC	GAAGATTCGTCGTGAAAGTCG

TNF-α, tumor necrosis factor-alpha; IL-6, interleukin-6; IL-1β, interleukin-1 beta.

**Table 2:** IC_50_ of the isolated compounds at different concentrations (100, 50, 25, 12.5, and 3.25 µg/mL) on HepG2.

Compound	HepG2 IC_50_ [µg/mL]
Chrysophanol	34.22 ± 0.10
Curcumin	80.73 ± 0.05
Hesperidin	95.66 ± 0.85
Physcion	172.6 ± 0.59

The results are shown as % cell viability relative to negative control cells, *n* = 3. Cell viability was established by MTT assay.

**Table 3:** The interaction of docking the isolated compounds (1–4) with key amino acid residues in the binding sites of 4 MAPKs (P38 α, ERK2, JNK& MK3), in comparison to the co-crystallized ligand.

Compound	P38 α MAPK			ERK1/2			JNK1			MK3		
	FlexX Score	Amino Acids Interactions	Hyde Assessment/Predicted Ki	FlexX Score	Amino Acids Interactions	Hyde Assessment/Predicted Ki	FlexX Score	Amino Acids Interactions	Hyde Assessment/Predicted Ki	FlexX Score	Amino Acids Interactions	Hyde Assessment/Predicted Ki
Curcumin	−24.639	Gly110, Gly033 Met109, Thr106, Asp 168, Asn155, His107	−34/µM	−21.8	MET 108, Gln105, Glu33, Thr 110, Lys114	−28 /µM	−17.72	Asn 114, Met111, Lys 55, Ile32	−23 /µM	−24.63	Lys73, Met121, Glu119, Glu125	−36/ nM
Physcion	−27.99	Gly110, Met109, Thr106, Ala 111, His107	−33/µM	−22.6	Met 108, Gln 105, Lys 54	−23/ µM	−24.07	Glu 109, Met111, Ile32	−32 /µM	−27.99	Met121, Glu119, Thr186	−24/ µM
Chrysophanol	−27.65	Gly110, Met109, Thr106, Ala 111, His107	−32/µM	−22.5	Gln 105, Asp 106, Met 108, Lys54, Asp167, Tyr36, Gly34, Ile56	−17/ mM	−24.44	Glu 109, Met111, Ile32, Ala53	−30 /µM	−27.65	Met121, Glu119, leu50, leu173	−35 /µM
Hesperidin	−20.03	Gly110, Met109, Thr106, Asp 168, Ser 154	−38/nM	−21.9	Asp 106, Met108, Lys114, Asp111, Tyr36, Glu33, Asn15, Gln105, Ile31	−14/ mM	−13.12	Glu 109, Met111, Ile32, Ser34, Lys55	−24/ µM	−20.03	Glu125, Cys120, Met121, Asp187, Glu 170, Lys 73	−22/ µM
(Co-crystallized ligand)	−30.7	Gly110, Met109 Thr106, Ala 157	−41/nM	−39	Lys 54, Asp 167, Met 108, Thr 110, Tyr-36, Gly34, Ile56	−45/ nM	−29	Ile 32, Met111, Glu 109, Val158, Leu168, Ala53, Val40	−39/ nM	−43.17	Met121, Cys120, Glu 170, Glu 125, leu52	−18 /mM
